# Interannual Climatic Variability Modulates Biostimulant and Herbicide Effects on Yield and Seed Quality of White Lupin Under Rainfed Conditions

**DOI:** 10.3390/plants15050726

**Published:** 2026-02-27

**Authors:** Florin Velică, Ioan Puiu, Dănuț-Petru Simioniuc, Carmen-Simona Ghițău, Teodor Robu

**Affiliations:** Department of Plant Science, “Ion Ionescu de la Brad” Iasi University of Life Sciences, 700490 Iași, Romania; florin.velica@iuls.ro (F.V.); ioan.puiu@iuls.ro (I.P.); carmen.ghitau@iuls.ro (C.-S.G.); teodor.robu@iuls.ro (T.R.)

**Keywords:** grain legumes, yield stability, phenotypic plasticity, nitrogen remobilization, seed quality traits, climate–management interaction, factorial field experiment

## Abstract

White lupin (*Lupinus albus L*.) is increasingly promoted as a sustainable protein crop; however, the consistency of agronomic input responses under rainfed continental conditions remains insufficiently documented. This study evaluated the interannual consistency of a foliar amino acid-based biostimulant (Aminotop N) and herbicide-based weed control on morphological traits, grain yield, seed physical quality (thousand-seed weight and hectoliter mass), and seed nitrogen and crude protein concentration of white lupin (cv. Măriuca). A 2 × 2 factorial field experiment arranged in a randomized complete block design was conducted across two consecutive growing seasons (2024–2025). Two-way ANOVA revealed significant effects of year and significant year × treatment interactions for grain yield and seed physical traits (*p* ≤ 0.05), indicating season-dependent treatment responses. Statistically significant treatment differences were detected in 2024, whereas no differences were observed among treatments in 2025. Seed total nitrogen concentration and crude protein content remained statistically stable across treatments and years. Principal component analysis explained 98.2% of total variance, with the first component primarily separating years rather than treatments. These results demonstrate that treatment effects were not consistently expressed across seasons and highlight the necessity of multi-year validation when assessing agronomic input effectiveness in white lupin under rainfed conditions.

## 1. Introduction

The increasing demand for sustainable plant protein sources has intensified interest in grain legumes as key components of resilient agricultural systems. Grain legumes contribute to agroecosystem sustainability through biological nitrogen fixation (BNF), reduced reliance on synthetic nitrogen fertilizers, and improved nitrogen cycling in cropping systems [1,2,3].

Among temperate grain legumes, white lupin (*L. albus*) is recognized for its high seed protein concentration, favorable amino acid profile, and adaptability to constrained soils [4,5,6]. Its capacity to develop specialized root adaptations and tolerate edaphic limitations further supports its agronomic potential under low-input conditions [5,6].

Yield formation and seed physical traits in white lupin are strongly influenced by environmental conditions during flowering (BBCH 60–69) and seed filling (BBCH 70–79) [7,8]. Seasonal climatic variability, particularly fluctuations in precipitation and temperature during reproductive development, can substantially affect assimilate allocation and final yield components [7,8]. Consequently, treatment responses observed in single-season field trials may not be consistently expressed across years.

Plant biostimulants, including amino acid-based formulations and protein hydrolysates, have been proposed as tools to enhance nutrient assimilation, metabolic activity, and stress tolerance [9,10]. However, responses in grain legumes remain variable and highly context-dependent, often influenced by environmental conditions and crop physiological status [8,10]. In white lupin specifically, field studies evaluating biostimulant applications have reported inconsistent effects on growth and yield performance [11].

Weed management represents another critical component in white lupin production due to its relatively slow early development and sensitivity to competition. Fluazifop-P-butyl, an aryloxyphenoxypropionate herbicide commonly used for post-emergence grass weed control, acts primarily through inhibition of acetyl-CoA carboxylase (ACCase), a key enzyme involved in fatty acid biosynthesis in susceptible species [12]. Herbicide-based weed control is commonly employed; however, crop tolerance and productivity responses may vary depending on environmental conditions and active ingredients [13]. Previous studies have demonstrated that herbicide treatments can influence plant development and yield performance in sweet white lupin under specific agro-climatic contexts [13].

Despite increasing adoption of both biostimulants and herbicide-based strategies in grain legume production, factorial multi-year field evaluations simultaneously assessing their combined effects under rainfed continental conditions remain limited. Most available studies rely on single-season assessments [11,13], restricting the ability to distinguish consistent management effects from year-specific climatic modulation.

Therefore, the objective of the present study was to evaluate the consistency of biostimulant and herbicide effects on morphological traits, grain yield, seed physical quality (thousand-seed weight and hectoliter mass), and seed nitrogen and crude protein content of white lupin (*L. albus*, cv. Măriuca) across two consecutive growing seasons under identical rainfed management conditions.

## 2. Results

### 2.1. Climatic Conditions During the Experimental Period

The two growing seasons (2024 and 2025) differed markedly in precipitation distribution and temperature patterns, particularly in relation to key phenological stages of white lupin. Under rainfed conditions, not only total seasonal rainfall but also its temporal alignment with flowering (BBCH 60–69) and seed filling (BBCH 70–79) is critical for yield formation and seed development.

**Figure 1:**
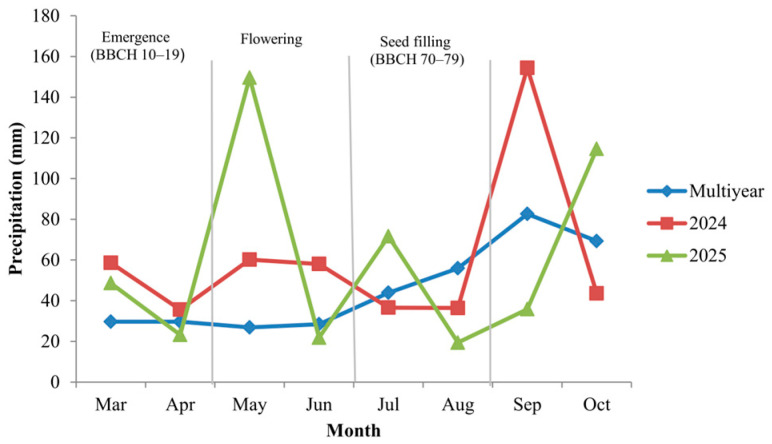
Monthly precipitation (mm) during the 2024 and 2025 growing seasons compared with the 1991–2020 long-term mean. Vertical lines indicate major phenological stages of white lupin (emergence BBCH 10–19, flowering BBCH 60–69, seed filling BBCH 70–79).

In 2024, precipitation was relatively adequate during early vegetative stages (BBCH 10–19), followed by reduced rainfall during flowering and early seed filling. In contrast, the 2025 growing season exhibited comparatively lower precipitation during early vegetative development but more favorable rainfall distribution during reproductive stages (Figure 1). These differences are agronomically relevant because reproductive stages represent a critical window for assimilate allocation and seed set determination.

**Figure 2:**
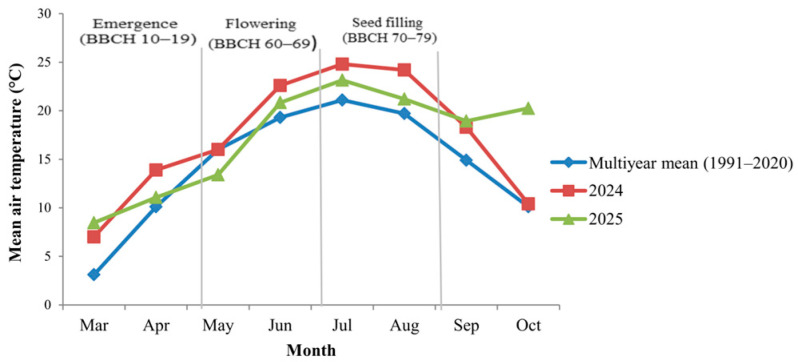
Monthly mean air temperature (°C) during the 2024 and 2025 growing seasons compared with the 1991–2020 long-term mean. Vertical lines indicate major phenological stages of white lupin.

Mean air temperature patterns also differed between the two years (Figure 2). In 2024, elevated temperatures coincided with the reproductive phase, particularly during early seed filling, whereas in 2025 temperature values during flowering were closer to the long-term mean. Such phenology-aligned climatic contrasts provide the environmental framework for interpreting the year-dependent responses observed for yield and seed physical traits.

### 2.2. Morphological Traits

Morphological traits of white lupin differed between the two growing seasons. Clear interannual differences were observed for plant height, first pod insertion, number of pods per plant, number of seeds per plant, pod length, pod width, and seed weight per plant.

In 2024, statistically significant differences among treatments were detected for several morphological traits (Table 1). In contrast, during the 2025 growing season, no statistically significant differences among treatments were identified within the experimental variability.

The combined results for both years are presented in Table 1.

### 2.3. Grain Yield

Two-way ANOVA performed on plot-level data (*n* = 24) revealed significant effects of Year (F_1_,_16_ = 55.61, *p* < 0.001) and Treatment (F_3_,_16_ = 7.90, *p* = 0.0019), and a significant Year × Treatment interaction (F_3_,_16_ = 8.06, *p* = 0.0017) for grain yield (Table S2). The presence of a significant interaction indicates that treatment responses differed between growing seasons.

**Figure 3:**
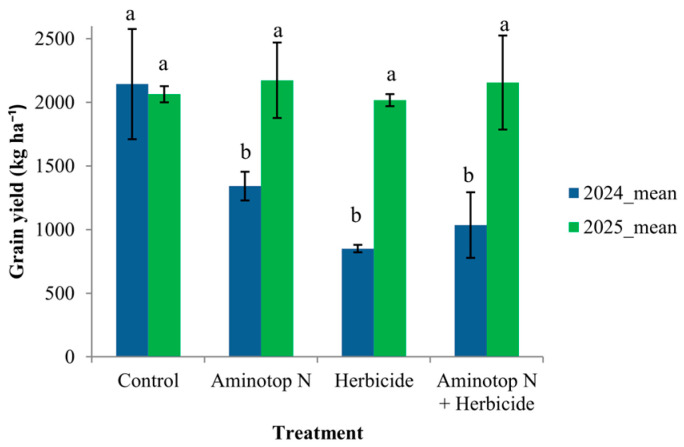
Interaction between year and treatment for grain yield of white lupin (*L. albus*, cv. Măriuca) under rainfed conditions. Values represent plot-based means ± SD (*n* = 3). Different letters indicate significant differences among treatments within the same year according to Tukey’s HSD test (*p* ≤ 0.05).

In the 2024 growing season, statistically significant differences among treatments were detected according to Tukey’s HSD test (*p* ≤ 0.05) (Figure 3). Grain yield ranged from 850 to 2144 kg ha^−1^, with the control treatment recording the highest mean value. In contrast, during the 2025 growing season, no statistically significant differences among treatments were observed, and grain yield ranged from 2017 to 2174 kg ha^−1^.

These results indicate that interannual climatic conditions modulated treatment responses, with detectable treatment effects expressed only in 2024, whereas under the climatic conditions of 2025 the tested input combinations resulted in comparable yield performance.

### 2.4. Thousand-Seed Weight and Hectoliter Mass

For thousand-seed weight (TSW), two-way ANOVA revealed a highly significant effect of Year (F_1_,_16_ = 434.87, *p* < 0.001) and a significant Year × Treatment interaction (F_3_,_16_ = 8.86, *p* = 0.0011), whereas the main effect of Treatment was not statistically significant (F_3_,_16_ = 1.52, *p* = 0.2380) (Table S1). Similarly, for hectoliter mass (HM), Year exerted a significant effect (F_1_,_16_ = 528.66, *p* < 0.001), and a significant Year × Treatment interaction was detected (F_3_,_16_ = 3.83, *p* = 0.0300), while the main effect of Treatment was not significant (F_3_,_16_ = 1.59, *p* = 0.2130).

In 2024, statistically significant differences among treatments were observed for both TSW and HM (Table 2). The combined application of biostimulant and herbicide resulted in lower mean TSW and HM compared with the control and single-input treatments. In contrast, during the 2025 growing season, neither TSW nor HM differed significantly among treatments within the experimental variability.

The strong year effect and significant interaction indicate that seed physical traits were primarily influenced by interannual climatic conditions, while treatment effects were expressed only under specific seasonal contexts.

### 2.5. Total Nitrogen and Crude Protein Content

Seed total nitrogen concentration and crude protein content did not differ significantly among treatments in either growing season.

In 2024, total nitrogen ranged from 5.68% to 6.04%, corresponding to crude protein values between 35.50% and 37.75% (Table 3). In 2025, nitrogen concentration ranged from 6.11% to 6.41%, with crude protein values between 38.19% and 40.06%. No statistically significant treatment effects were detected within each year.

### 2.6. Principal Component Analysis

Principal component analysis (PCA) was performed using plot-level observations (*n* = 24) to integrate grain yield, thousand-seed weight, and hectoliter mass into a multivariate framework.

**Figure 4:**
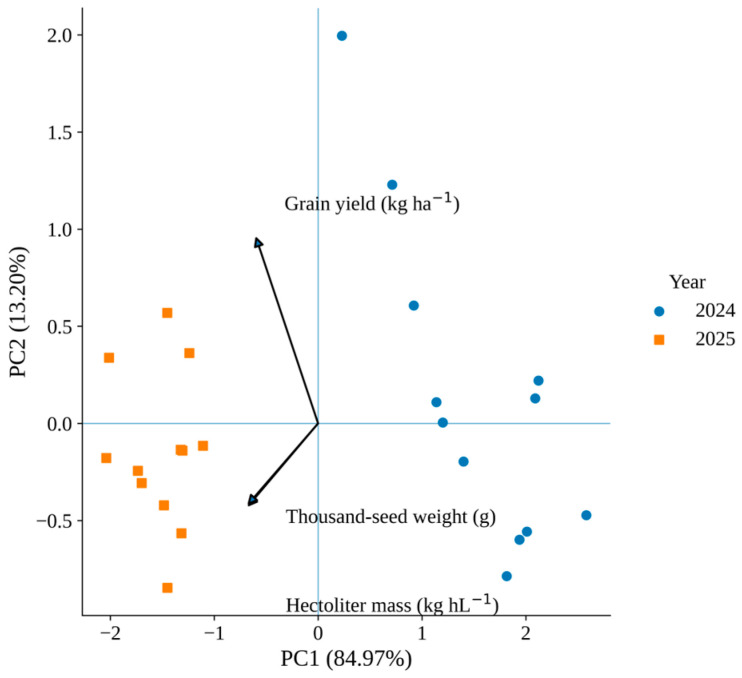
Principal component analysis (PCA) biplot of grain yield, thousand-seed weight (TSW), and hectoliter mass (HM) across the 2024 and 2025 growing seasons. PC1 explains 84.97% and PC2 13.20% of total variance. Points represent plot-level observations (*n* = 24). Variables were standardized prior to analysis. Arrows indicate the direction and magnitude of variable loadings.

The first principal component (PC1) explained 84.97% of the total variance, while the second principal component (PC2) accounted for 13.20%, resulting in a cumulative variance of 98.17%. PC1 was primarily associated with yield and seed physical traits and separated the two growing seasons along a productivity gradient (Figure 4).

Plot-level observations clustered distinctly according to year, while treatment-level separation within each year was limited. The PCA configuration indicates that interannual differences contributed more strongly to trait variability than the tested input combinations.

### 2.7. Pearson Correlation Analysis

**Figure 5:**
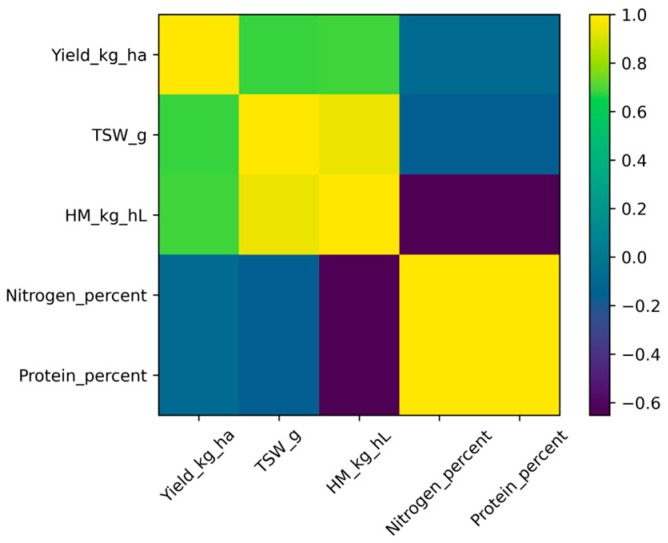
Pearson correlation matrix among grain yield (Yield_kg_ha), thousand-seed weight (TSW_g), hectoliter mass (HM_kg_hL), total nitrogen concentration (N%), and crude protein content based on plot-level observations (*n* = 24). Color scale represents Pearson correlation coefficients (r). Crude protein values are derived from total nitrogen concentration using a fixed conversion factor (protein = N × 6.25).

Pearson correlation analysis was performed using plot-level observations (*n* = 24) to evaluate linear relationships among grain yield, thousand-seed weight (TSW), hectoliter mass (HM), total nitrogen concentration, and crude protein content (Figure 5).

Grain yield showed a strong positive correlation with thousand-seed weight (r = 0.676) and hectoliter mass (r = 0.691). Thousand-seed weight and hectoliter mass were highly correlated (r = 0.949), indicating close association between seed size and bulk density traits.

Total nitrogen concentration was not correlated with grain yield (r = −0.079). A moderate negative correlation was observed between hectoliter mass and nitrogen concentration (r = −0.647). Crude protein content exhibited identical correlation patterns to total nitrogen concentration, reflecting its derivation from nitrogen concentration using a fixed conversion factor (protein = N × 6.25).

These results indicate that variation in grain yield was primarily associated with seed physical traits rather than seed nitrogen concentration, supporting the separation between carbon-driven yield formation and nitrogen-buffered compositional traits.

## 3. Discussion

### 3.1. Climatic Modulation of Treatment Responses

Two-way ANOVA revealed significant year and year × treatment interactions for grain yield and seed physical traits, indicating that treatment responses were season-dependent. In temperate rainfed systems, reproductive development in grain legumes is highly sensitive to environmental conditions during flowering and early seed filling [7,8]. Variations in temperature and water availability during these stages can modify assimilate supply and reproductive sink strength, thereby influencing the magnitude of management effects.

White lupin exhibits adaptive plasticity in reproductive allocation under variable environments [7]. When environmental conditions support stable assimilate supply, differentiation among management variants may remain limited. Conversely, under more restrictive conditions, reduced buffering capacity may increase the detectability of treatment-related differences.

### 3.2. Morphological Trait Responses Under Variable Seasonal Conditions

Morphological traits in white lupin reflect the integration of vegetative growth and reproductive development under prevailing environmental conditions. Traits such as plant height, first pod insertion height, and pod number are influenced by resource availability during early growth stages and by assimilate distribution during the transition to reproductive development [7]. Interannual variability in these traits is therefore consistent with the documented sensitivity of lupin canopy architecture to climatic factors, particularly water availability and temperature regime.

In grain legumes, reproductive components including pods per plant and seeds per plant are frequently more responsive to environmental variation than to moderate agronomic interventions [8]. Fluctuations in temperature and soil moisture during stem elongation and flowering may influence branching intensity, flower retention, and pod set, thereby affecting final yield structure. Such environmentally driven adjustments contribute to the plasticity of morphological expression observed across growing seasons.

In the present study, the absence of consistent treatment effects on morphological traits across both experimental years indicates that short-term biostimulant or herbicide applications did not induce stable structural modifications under the evaluated conditions. Instead, seasonal context appears to have played a dominant role in shaping canopy development and reproductive architecture, in agreement with previous observations in white lupin and other grain legumes cultivated under rainfed systems [7].

Morphological plasticity in white lupin has been described as an adaptive strategy enabling modulation of reproductive effort under contrasting environmental scenarios [8]. This flexibility may attenuate management-driven contrasts under favorable conditions while amplifying them under environmental constraints. Consequently, interpretation of treatment effects on structural traits in multi-year field experiments should account for the interaction between developmental plasticity and seasonal climatic variability.

### 3.3. Grain Yield Responses and Assimilate Allocation

Grain yield in white lupin is primarily determined during the reproductive period, particularly between flowering (BBCH 60–69) and early seed filling (BBCH 70–79), when pod set and seed growth are highly sensitive to environmental conditions. In temperate rainfed systems, fluctuations in temperature and precipitation during these stages can alter assimilate availability and reproductive sink establishment, thereby influencing final yield expression [8].

In addition to environmental influences, the physiological mode of action of herbicide treatments may also contribute to crop responses. Fluazifop-P-butyl, an aryloxyphenoxypropionate graminicide, acts primarily through inhibition of acetyl-CoA carboxylase (ACCase), a key enzyme involved in de novo fatty acid biosynthesis in susceptible species [12]. Although white lupin is a dicotyledonous crop and therefore tolerant to ACCase-inhibiting herbicides, transient metabolic adjustments following herbicide exposure under field conditions cannot be entirely excluded. Environmental stress factors, particularly drought and elevated temperatures, may further modulate plant recovery capacity after application, potentially contributing to variability in treatment responses observed across seasons.

In the present study, significant Year and Year × Treatment effects indicate that treatment responses were not stable across contrasting seasonal contexts. The separation of years observed in the PCA suggests that interannual climatic variability represented the dominant source of yield-related variation. These patterns are consistent with previous reports indicating that grain legumes frequently exhibit stronger year effects than management effects under rainfed conditions [7]. In rainfed cropping systems, periods characterized by elevated temperatures combined with reduced precipitation can limit physiological activity, nutrient uptake, and assimilate partitioning in grain legumes. Under such stress conditions, plant responses to agronomic inputs may be attenuated or delayed, resulting in reduced or non-significant treatment effects despite similar management practices. Therefore, the absence of detectable treatment differences in certain conditions may reflect environmental constraints rather than a lack of treatment efficacy. Monthly climatic indicators aligned with crop phenological stages provided sufficient environmental context for interpreting seasonal variability. Analysis of daily meteorological data recorded during the two weeks following herbicide and biostimulant application further revealed substantial thermal contrasts between experimental years (Table S3). In 2024, mean daily air temperature during the post-application period averaged approximately 21.9 °C, with frequent maximum temperatures exceeding 30 °C. In contrast, the corresponding period in 2025 was characterized by markedly lower temperatures, averaging approximately 13.8 °C, with maximum values generally below 26 °C. These thermal differences indicate that plants experienced substantially higher heat load following treatment application in 2024 compared with 2025, which may have influenced physiological recovery capacity and contributed to the interannual variability observed in treatment responses. This pattern is consistent with the comparatively more favorable environmental conditions during reproductive development in 2025, which likely reduced physiological stress intensity and consequently limited the expression of treatment-related contrasts.

Yield differences observed in 2024, but not in 2025, may reflect differential environmental constraints during key reproductive stages. Elevated temperatures or reduced precipitation during flowering and early seed filling can limit assimilate supply and reproductive retention, potentially increasing the detectability of treatment-related differences. Conversely, when environmental conditions are closer to optimal during reproductive development, crop physiological buffering capacity may reduce apparent management contrasts.

In addition to direct climatic influences on canopy physiology, indirect effects mediated through soil processes may contribute to interannual variability. Soil moisture fluctuations can affect nutrient mobility, root activity, and microbial processes involved in biological nitrogen fixation, thereby indirectly influencing reproductive performance. Although direct measurements of carbon fluxes, nitrogen fixation rates, or root activity were not conducted in the present study, the observed yield patterns are compatible with established source–sink regulatory frameworks described for grain legumes [1,8].

Therefore, the interannual modulation of treatment effects observed here should be interpreted as environmentally mediated rather than as evidence of consistent agronomic response. These findings highlight the importance of multi-year validation when evaluating input effectiveness in white lupin under rainfed continental conditions.

### 3.4. Seed Physical Quality: Thousand-Seed Weight and Hectoliter Mass

Thousand-seed weight and hectoliter mass represent integrative indicators of seed filling efficiency and reflect the balance between assimilate supply and reproductive sink demand. In grain legumes, final seed mass depends on the duration and stability of carbon allocation during seed filling, a phase highly sensitive to environmental conditions [10]. Variations in temperature and water availability during this period can influence both the rate and duration of dry matter accumulation in developing seeds.

Compared with compositional parameters such as protein concentration, seed physical traits often exhibit greater responsiveness to environmental variability. Changes in assimilate availability may directly affect seed size and bulk density without proportionally modifying nitrogen partitioning patterns. This differential sensitivity may explain why thousand-seed weight and hectoliter mass displayed significant year and interaction effects, whereas nitrogen concentration remained comparatively stable.

In white lupin, seed development depends on coordination between vegetative source capacity and developing reproductive sinks. Under conditions of stable assimilate supply, seed filling may proceed with limited variation among treatment combinations. Conversely, when carbon availability is constrained, even moderate shifts in source–sink balance may influence final seed mass.

The pattern observed in the present study aligns with the known physiological regulation of seed filling in legumes, where environmental modulation of carbon fluxes frequently exceeds the influence of short-term agronomic inputs [10]. Therefore, interpretation of treatment effects on seed physical quality under rainfed conditions requires consideration of seasonal assimilate dynamics rather than isolated evaluation of applied formulations.

### 3.5. Interpretation of the Biostimulant Composition

Amino acid-based biostimulants and protein hydrolysates have been reported to influence plant metabolism and stress-related responses through multiple pathways, including nutrient assimilation and carbon–nitrogen interactions; however, responses are strongly context-dependent and environmentally modulated [9,10].

Because Aminotop N contains several bioactive fractions, the present factorial field experiment does not allow attribution of observed responses to a specific component. The results therefore reflect the agronomic performance of the commercial formulation as a whole.

### 3.6. Seed Nitrogen and Crude Protein Stability

Seed nitrogen concentration and crude protein content in grain legumes are regulated by the interaction between biological nitrogen fixation, soil nitrogen availability, and internal nitrogen remobilization processes [1,2,3]. In white lupin, symbiotic nitrogen fixation represents a major source of nitrogen supply under low-input rainfed conditions, contributing substantially to seed nitrogen accumulation [1,2,3].

Unlike yield and seed mass traits, which are directly linked to carbon availability during seed filling, nitrogen concentration is influenced by ongoing fixation activity and redistribution of stored nitrogen from vegetative tissues to developing seeds [1,3]. These physiological processes may buffer seed compositional traits against moderate environmental fluctuations.

Environmental variation during reproductive development may reduce overall biomass production without proportionally altering nitrogen concentration when nitrogen fixation and remobilization remain functional [1,2,3]. This regulatory capacity can result in relatively stable protein levels even when yield components vary between seasons.

The stability of seed nitrogen and crude protein content observed in the present study is therefore consistent with the established role of biological nitrogen fixation and internal nitrogen dynamics in legumes [1,2,3], which may reduce the sensitivity of compositional traits to short-term agronomic interventions compared with carbon-dependent parameters such as seed mass [10].

### 3.7. Multivariate Relationships Among Yield and Seed Traits

Multivariate analysis was applied to explore coordinated relationships among yield and seed quality traits beyond simple pairwise comparisons. Principal component analysis (PCA) integrated grain yield, thousand-seed weight, and hectoliter mass into a unified framework, revealing that interannual variation represented the dominant source of total variability.

Traits associated with yield and seed physical quality clustered closely, reflecting coordinated variation likely linked to assimilate allocation during reproductive development [10]. In contrast, total nitrogen concentration and crude protein content showed weaker association with yield-related traits, indicating partial independence between carbon-driven yield formation and nitrogen metabolism [1,8].

This separation in multivariate space reflects the distinct physiological regulation of carbon fluxes and nitrogen partitioning in legumes [1,8]. While yield and seed mass are primarily influenced by source–sink balance and carbon availability during seed filling, nitrogen concentration is supported by biological fixation and remobilization mechanisms [1,2,3].

Pearson correlation analysis further supported this interpretation, showing strong positive relationships among yield and seed mass traits, alongside limited association between yield and nitrogen concentration. Such patterns are consistent with previously described functional differentiation between carbon-dependent and nitrogen-buffered traits in grain legumes [1,8].

The present study did not include direct physiological or biochemical measurements; therefore, mechanistic explanations remain limited to interpretation grounded in established physiological frameworks [1,2,3,8]. Elucidation of specific regulatory processes underlying year-dependent treatment responses would require future integration of physiological indicators.

## 4. Materials and Methods

### 4.1. Study Site

The field experiments were conducted during the 2024 and 2025 growing seasons at the Teaching and Research Station Ezăreni of the University of Life Sciences “Ion Ionescu de la Brad” Iași, Romania (47°07′25″ N, 27°31′01″ E), located in the southwestern sector of the Moldavian Plain (Lower Jijia Plain).

The predominant soil type at the experimental site is a cambic chernozem, classified according to the World Reference Base (WRB), with a loam to clay-loam texture. The soil reaction was slightly acidic, with pH (H_2_O) 6.8. The humus content in the 0–20 cm layer was 2.79%. Baseline soil physico-chemical properties were derived from an institutional agrochemical soil survey conducted in 2021 by the Research Institute for Agriculture and Environment Iași. According to this survey, total nitrogen content was 0.198 g·100 g^−1^ soil, mobile phosphorus was 1.2 mg P_2_O_5_·100 g^−1^ soil, and mobile potassium was 11.7 mg K_2_O·100 g^−1^ soil. Soil pH was determined according to SR ISO 10390 [14], humus content by the Walkley–Black method, and available phosphorus and potassium by the Egner–Riehm–Domingo method.

The preceding crop was winter wheat in 2024 and maize (*Zea mays* L.) in 2025. Conventional tillage was applied in both years, consisting of autumn plowing followed by spring seedbed preparation prior to sowing.

### 4.2. Plant Material

The plant material used in this study was white lupin (*L. albus*, cv. Măriuca), a Romanian cultivar officially registered in the National Catalogue of Cultivated Plant Varieties of Romania and developed at the University of Life Sciences “Ion Ionescu de la Brad” Iași for grain production. The cultivar is classified as sweet white lupin, characterized by the absence of bitter alkaloids in the seeds and a determinate growth habit.

According to the official cultivar description, Măriuca exhibits medium to tall plant stature and is adapted to cultivation under rainfed conditions. The reported thousand-seed weight in the official catalogue is approximately 329 g [3].

Seed material was supplied by the University of Life Sciences “Ion Ionescu de la Brad” Iași and originated from seed multiplication conducted during the 2023 growing season. Prior to sowing, seed physical purity and germination capacity were determined under laboratory conditions in accordance with International Seed Testing Association (ISTA) rules. Physical purity was assessed by gravimetric separation of pure seeds from impurities, while germination capacity was determined using standard germination tests and expressed as the percentage of normal seedlings.

The seed lot used in the experiment exhibited a physical purity of 98% and a germination capacity of 95% and was not subjected to chemical or biological seed treatment prior to sowing.

### 4.3. Experimental Design

The field experiment was established as a randomized complete block design (RCBD) arranged in a 2 × 2 factorial scheme. The two experimental factors were:

- Biostimulant application (absence vs. application of Aminotop N).

- Weed control (absence vs. herbicide application). The factorial combination of these factors resulted in four treatments: untreated control, biostimulant only, herbicide only, and combined biostimulant and herbicide application.

Each treatment was replicated three times in each experimental year. Experimental blocks were arranged along the main field slope (2–3%) to reduce the influence of soil heterogeneity and microtopographic variation. Within each block, treatments were randomly assigned to plots using a simple randomization procedure.

Each experimental plot had a gross area of 6 m^2^. All quantitative measurements and yield determinations were performed on a centrally located net plot area of 2 m^2^, excluding border rows to minimize edge effects. The experimental unit was defined as the plot.

With four treatments and three replications per year, the experiment comprised 12 plots annually and a total of 24 experimental units across the two-year study period.

The preceding crop was winter wheat in 2024 and maize (*Zea mays* L.) in 2025. The field layout was re-established each year, and year was considered as an additional fixed factor in the statistical analysis.

### 4.4. Crop Management

Crop management practices were identical in both experimental years, with differences limited to calendar dates determined by prevailing weather conditions. The soil was ploughed in autumn (2023 and 2024) and disked in early spring, and the seedbed was prepared one day prior to sowing. The crop was grown under rainfed conditions without the application of mineral fertilizers, insecticides, or fungicides.

Sowing was performed manually at a density of 30 viable seeds m^−2^ with a row spacing of 0.50 m, on 5 April 2024 and 26 March 2025. Crop phenological development and the timing of all management operations were monitored according to the extended BBCH scale for mono- and dicotyledonous plants [15].

In the herbicide-treated variants, pre-emergence weed control was achieved using Challenge 600 SC (Bayer Crop Science, Monheim am Rhein, Germany; active ingredient: aclonifen, 600 g L^−1^), applied at a rate of 4 L ha^−1^ (2400 g a.i. ha^−1^) on the day of sowing (BBCH 00). Post-emergence grass weed control was performed using Fusilade Forte (Syngenta Crop Protection AG, Basel, Switzerland; active ingredient: fluazifop-P-butyl, 150 g L^−1^), applied at 1 L ha^−1^ (150 g a.i. ha^−1^) at BBCH stages 13–14 (three to four true leaves), on 11 June 2024 and 15 May 2025.

All chemical applications were performed using a 15 L backpack sprayer equipped with hollow-cone nozzles (Solo Kleinmotoren GmbH, Sindelfingen, Germany), calibrated to deliver a spray volume of 300 L ha^−1^.

In non-herbicide treatments, weed control was performed mechanically through two inter-row cultivations per growing season.

The biostimulant Aminotop N was applied at a rate of 1 L ha^−1^ at BBCH stages 14–16 (four to six true leaves), two days after the post-emergence herbicide application in the corresponding treatments. Aminotop N is a foliar plant biostimulant based on free amino acids and organically bound nitrogen, manufactured by Timac Agro (Groupe Roullier, Saint-Malo, France) and classified as a plant biostimulant under Regulation (EU) 2019/1009. The product was evaluated as a commercial multi-component formulation. Therefore, the present experimental design does not allow separation of the individual contribution of specific fractions (e.g., free amino acids, humic substances, or seaweed extract). Observed responses should be interpreted at the formulation level rather than attributed to a single active component. No visible phytotoxicity symptoms were observed following herbicide or biostimulant applications.

The declared chemical composition of Aminotop N used in the experiment is presented in Table 4.

### 4.5. Climate Data

Meteorological data were obtained from an automated iMetos weather station (Pessl Instruments GmbH, Weiz, Austria) installed at the Teaching and Research Station Ezăreni, approximately 400 m from the experimental field, with continuous recording of standard agrometeorological parameters using calibrated sensors. Daily air temperature (°C) and precipitation (mm) data were aggregated into monthly mean air temperatures and monthly total precipitation values for the 2024 and 2025 growing seasons. Long-term reference values (1991–2020) were calculated in accordance with the *Guide to Climatological Practices* (WMO-No. 100) of the World Meteorological Organization [16]. Monthly precipitation totals and monthly mean air temperatures for both experimental years, together with corresponding long-term averages, are presented in Figure 1 and Figure 2. The monthly climatic data used to generate these figures are provided in Supplementary Materials (Table S1). Climatic variables were used solely to describe environmental conditions during crop development and were not included as independent variables in the statistical analyses. Daily air temperature data recorded during the post-application period were additionally analyzed to support the interpretation of treatment responses and are provided in Supplementary Materials (Table S3).

### 4.6. Morphological and Yield Measurements

Morphological and yield-related traits were assessed at the plot level for each biological replication. Plot means were considered as experimental units and were used in all statistical analyses.

At physiological maturity, plants from the net plot area (2 m^2^) were harvested manually for grain yield determination. After cleaning and weighing, plot yield was recorded in kilograms and converted to *yield* per hectare (kg ha^−1^) using the following equation:

$$Yield\;(kg\;{ha}^{-1})=\frac{Plot\;yield\;(kg)}{Harvested\;area\;(m^{2})}\times 10,000$$

For morphological characterization, representative plants were sampled from each plot. Plant height (cm) was measured from the soil surface to the apex of the main stem, and the height of first pod insertion (cm) was measured from the soil surface to the lowest pod. The number of pods per plant and seeds per plant were determined by manual counting at harvest.

Seed weight per plant was measured using a precision laboratory balance (IoT-Line, KERN & Sohn GmbH, Balingen, Germany; readability 0.1 g).

Thousand-seed weight (TSW) was determined by weighing 1000 seeds randomly selected from the harvested grain of each plot, following the procedures described in the International Rules for Seed Testing [17].

Hectoliter mass (HM) was determined using a portable 0.5 L hectoliter apparatus (Pfeuffer GmbH, Kitzingen, Germany) and expressed as kg hL^−1^. Measurements were performed in accordance with ISO 7971-1:2019 [18]. Although the standard is primarily intended for cereals, it is commonly applied in agronomic research for comparative assessment of bulk density in large-seeded legumes.

### 4.7. Biochemical Analyses

Total nitrogen content in white lupin seeds was determined using the Kjeldahl method in accordance with ISO 1871:2009 [19] and AOAC Official Method 988.05 [20].

Seed samples were collected at full maturity, cleaned of impurities, and ground to a homogeneous powder using a laboratory knife mill (GM200, Retsch GmbH, Haan, Germany). Prior to analysis, seed moisture content was determined by oven-drying at 105 °C to constant weight, and all results were expressed on a dry matter basis.

For each treatment and biological replication, 1.0 g of ground seed material was subjected to wet acid digestion with concentrated sulfuric acid (H_2_SO_4_) in the presence of a Kjeldahl catalyst mixture (K_2_SO_4_–CuSO_4_) until complete mineralization. After digestion, samples were diluted and rendered alkaline with sodium hydroxide, and the released ammonia was distilled and collected in a boric acid solution with an appropriate indicator.

The distilled ammonia was quantified by volumetric titration, and total nitrogen concentration was calculated based on titrant consumption and expressed as percentage nitrogen (% N) on a dry matter basis. One analytical determination was performed for each biological replication.

Crude protein content was calculated from total nitrogen concentration using the conventional nitrogen-to-protein conversion factor for legume seeds:

Crude protein (%) = N (%) × 6.25

### 4.8. Statistical Analysis

Experimental data were analyzed using Microsoft Excel and Python (version 3.13.9, 64-bit). Data organization and descriptive statistics were performed using the *pandas* and NumPy libraries.

Prior to inferential analyses, model assumptions were evaluated. Normality of residuals was assessed using the Shapiro–Wilk test [21], and homogeneity of variances was tested using the Brown–Forsythe modification of Levene’s test [22]. Assumptions were considered satisfied when *p*-values were greater than 0.05.

For each trait, a two-way analysis of variance (ANOVA) was conducted according to the 2 × 2 factorial design described in Section 4.3. Year (Y), treatment (T), and their interaction (Y × T) were treated as fixed effects, following standard procedures for factorial experimental designs in agronomic research [23].

The statistical model applied was:

Y_ijk_ = μ + Y_i_ + T_j_ + (Y × T)_ij_ + ε_ijk_

where μ represents the overall mean;

Y_i_ the effect of the i-th year;

T_j_ the effect of the j-th treatment;

(Y × T)_ij_ the interaction effect;

ε_ijk_ the residual error term.

Plot-level values (*n* = 24) were defined as experimental units for all statistical analyses. When the Year × Treatment interaction was not significant (*p* > 0.05), treatment main effects were interpreted across years. When a significant interaction was detected (*p* ≤ 0.05), treatment effects were analyzed separately within each experimental year. Mean comparisons were performed using Tukey’s honestly significant difference (HSD) test [24] at a significance level of *p* ≤ 0.05.

Principal component analysis (PCA) was performed using standardized plot-level data (*n* = 24) to examine multivariate relationships among grain yield, thousand-seed weight, and hectoliter mass [25].

Pearson’s correlation coefficients (r) were calculated using plot-level observations (*n* = 24) to evaluate linear relationships among grain yield, thousand-seed weight, hectoliter mass, total nitrogen concentration, and crude protein content [26].

### 4.9. Limitations of the Study

The present study was conducted under field conditions and focused on agronomic and seed quality parameters. No direct physiological, enzymatic, or molecular measurements were performed; therefore, mechanistic interpretation of treatment effects remains limited to phenotypic and statistical evidence. In addition, the experimental period covered two consecutive growing seasons under rainfed conditions, which constrains extrapolation beyond similar climatic environments. Future research integrating controlled physiological assays and multi-location trials would provide deeper insight into treatment-specific mechanisms and environmental stability.

## 5. Conclusions

Significant Year and Year × Treatment effects were identified for grain yield, thousand-seed weight, and hectoliter mass in the two-year factorial experiment conducted under rainfed conditions. Treatment-related differences were detectable in 2024 but not in 2025, demonstrating season-dependent responses.

Seed total nitrogen concentration and crude protein content remained statistically stable across treatments and years.

Principal component analysis confirmed that interannual variation contributed more strongly to overall trait variability than the tested treatment combinations.

These findings indicate that short-term agronomic input evaluations may not provide consistent conclusions under temperate rainfed systems and highlight the necessity of multi-year factorial validation in white lupin.

## Figures and Tables

**Table 1:** Effects of biostimulant and herbicide treatments on morphological traits of white lupin (*L. albus*, cv. Măriuca) across two growing seasons (2024–2025).

Year	Treatment	Plant Height (cm)	First Pod Insertion (cm)	Pods Per Plant	Seeds Per Plant	Pod Length (cm)	Pod Width (cm)	Seed Weight Per Plant (g)
2024	Control	78.00 ± 1.31^b^	49.97 ± 0.47 ^a^	5.55 ± 0.17^a^	19.93 ± 0.38^a^	7.20 ± 0.22 ^a^	1.30 ± 0.05 ^a^	5.00 ± 0.32^a^
	Aminotop N	76.77 ± 1.61^b^	50.22 ± 1.59 ^a^	5.12 ± 0.30^a^	19.50 ± 0.77^a^	7.01 ± 0.13 ^a^	1.28 ± 0.02 ^a^	4.53 ± 0.18^a^
	Herbicide	83.26 ± 0.78^a^	53.14 ± 1.79 ^a^	5.17 ± 0.39^a^	11.80 ± 1.16^c^	4.98 ± 0.26 ^c^	1.08 ± 0.07 ^b^	2.90 ± 0.18^b^
	Aminotop N + Herbicide	71.70 ± 2.84^c^	48.67 ± 3.38 ^a^	5.31 ± 1.02^a^	15.18 ± 1.91^b^	5.60 ± 0.27 ^b^	1.18 ± 0.08 ^a^	3.49 ± 0.49^b^
2025	Control	63.35 ± 2.98^a^	32.93 ± 3.32 ^a^	8.99 ± 0.19^a^	34.61 ± 1.00^a^	7.33 ± 0.08 ^a^	1.32 ± 0.03 ^a^	10.07 ± 0.31^a^
	Aminotop N	63.82 ± 4.18^a^	34.19 ± 1.68 ^a^	9.85 ± 1.63^a^	37.30 ± 6.21^a^	7.25 ± 0.02 ^a^	1.30 ± 0.02 ^a^	10.60 ± 1.45^a^
	Herbicide	64.04 ± 1.04^a^	33.57 ± 1.18 ^a^	9.56 ± 0.95^a^	36.22 ± 4.30^a^	7.40 ± 0.07 ^a^	1.34 ± 0.03 ^a^	10.40 ± 1.40^a^
	Aminotop N + Herbicide	65.17 ± 2.28^a^	33.44 ± 0.37 ^a^	10.37 ± 2.04^a^	40.02 ± 4.22^a^	7.46 ± 0.22 ^a^	1.35 ± 0.06 ^a^	11.40 ± 1.21^a^

Values represent plot-based means ± SD (*n* = 3). Different letters within the same year and column indicate significant differences among treatments according to Tukey’s HSD test (*p* ≤ 0.05).

**Table 2:** Effects of biostimulant and herbicide treatments on thousand-seed weight (TSW) and hectoliter mass (HM) of white lupin (*L. albus*, cv. Măriuca) across the 2024 and 2025 growing seasons.

Year	Treatment	TSW (g)	HM (kg hL^−1^)
2024	Control	241.85 ± 4.74 a	67.12 ± 1.05 a
	Aminotop N	239.95 ± 4.00 a	68.31 ± 0.71 a
	Herbicide	238.02 ± 9.69 a	67.11 ± 1.31 a
	Aminotop N + Herbicide	221.80 ± 2.17 b	65.54 ± 0.17 b
2025	Control	302.07 ± 6.91 a	74.90 ± 1.29 a
	Aminotop N	285.87 ± 11.93 a	74.98 ± 0.45 a
	Herbicide	295.30 ± 9.85 a	75.07 ± 0.42 a
	Aminotop N + Herbicide	310.00 ± 3.55 a	75.52 ± 0.74 a

Values represent plot-based means ± SD (*n* = 3). Different letters within the same year and column indicate significant differences among treatments according to Tukey’s HSD test (*p* ≤ 0.05).

**Table 3:** Effects of biostimulant and herbicide treatments on total nitrogen concentration and crude protein content of white lupin (*L. albus*, cv. Măriuca) across the 2024 and 2025 growing seasons.

Year	Treatment	Total Nitrogen (%)	Crude Protein (%)
2024	Control	5.68 ± 0.22 ^a^	35.50 ± 1.38 ^a^
	Aminotop N	5.80 ± 0.29 ^a^	36.25 ± 1.81 ^a^
	Herbicide	5.87 ± 0.02 ^a^	36.69 ± 0.13 ^a^
	Aminotop N + Herbicide	6.04 ± 0.18 ^a^	37.75 ± 1.13 ^a^
2025	Control	6.12 ± 0.41 ^a^	38.25 ± 2.56 ^a^
	Aminotop N	6.11 ± 0.34 ^a^	38.19 ± 2.13 ^a^
	Herbicide	6.37 ± 0.13 ^a^	39.81 ± 0.81 ^a^
	Aminotop N + Herbicide	6.41 ± 0.17 ^a^	40.06 ± 1.06 ^a^

Values represent plot-based means ± SD (*n* = 3). Different letters within the same year and column indicate significant differences among treatments according to Tukey’s HSD test (*p* ≤ 0.05).

**Table 4:** Declared composition of Aminotop N used in the experiment (according to manufacturer specifications).

Component	Declared Content (%)
Free amino acids	30.0
Organic nitrogen (N)	4.0
Humic substances	2.0
Seaweed extract (Ascophyllum nodosum)	1.2
Other organic components (q.s.)	Not specified

*Notes:* Composition values are based on the manufacturer’s technical specification sheet. No independent laboratory verification of the product composition was performed in this study. q.s. indicates quantity sufficient; not specified by the manufacturer.

## Data Availability

The datasets generated and analyzed during the current study are available from the corresponding author upon reasonable request.

## Supplementary Materials

The following supporting information can be downloaded at: https://www.mdpi.com/article/10.3390/plants15050726/s1. Table S1: Monthly precipitation totals (mm) and mean air temperatures (°C) recorded during the growing seasons (March–October) of 2024 and 2025 at the Ezăreni experimental station, compared with the 1991–2020 long-term averages. Table S2: Two-way ANOVA results for grain yield, thousand-seed weight (TSW), and hectoliter mass (HM) based on plot-level observations (n = 24). Table S3: Daily mean and maximum air temperature during the 14-day period following treatment application in 2024 and 2025.

## Abbreviations

BNF	Biological nitrogen fixation
TSW	Thousand-seed weight
HM	Hectoliter mass
PCA	Principal component analysis
ANOVA	Analysis of variance

## References

[1] Ladha J.K., Peoples M.B., Krupnik T.J., Reddy P.M., Biswas J.C., Bennett A., Jat M.L. (2022). Biological nitrogen fixation and prospects for ecological intensification in cereal-based cropping systems. Field Crops Res..

[2] Vanlauwe B., Hungria M., Giller K.E. (2019). The role of legumes in the sustainable intensification of African smallholder agriculture: Lessons learnt and challenges for the future. Agric. Ecosyst. Environ..

[3] Rodríguez C., Carlsson G., Jensen E.S. (2020). Grain legume-cereal intercropping enhances the use of soil-derived and biologically fixed nitrogen in temperate agroecosystems: A meta-analysis. Eur. J. Agron..

[4] Pereira A., Ramos F., Sanches Silva A. (2022). Lupin (*Lupinus albus* L.) seeds: Balancing the good and the bad and addressing future challenges. Molecules.

[5] Annicchiarico P., de Buck A.J., Crosta M. (2023). White lupin adaptation to moderately calcareous soils: Phenotypic variation and genome-enabled prediction. Plants.

[6] Quiñones M.A., Lucas M.M., Pueyo J.J. (2022). Adaptive mechanisms make lupin a choice crop for acidic soils affected by aluminum toxicity. Front. Plant Sci..

[7] Reckling M., Döring T.F., Bergkvist G., Stoddard F.L., Watson C.A., Seddig S., Chmielewski F.-M., Bachinger J. (2018). Grain legume yields are as stable as other spring crops in long-term experiments across northern Europe. Agron. Sustain. Dev..

[8] Morin A., Maurousset L., Vriet C., Lemoine R., Doidy J., Pourtau N. (2022). Carbon fluxes and environmental interactions during legume development, with a specific focus on *Pisum sativum*. Physiol. Plant..

[9] Sible C.N., Seebauer J.R., Below F.E. (2021). Plant biostimulants: A categorical review, their implications for row crop production, and relation to soil health indicators. Agronomy.

[10] Sun W., Shahrajabian M.H., Kuang Y., Wang N. (2024). Amino acids biostimulants and protein hydrolysates in agricultural sciences. Plants.

[11] Niewiadomska A., Sulewska H., Wolna-Maruwka A., Ratajczak K., Waraczewska Z., Budka A. (2020). The influence of bio-stimulants and foliar fertilizers on yield, plant features, and the level of soil biochemical activity in white lupine (*Lupinus albus* L.) cultivation. Agronomy.

[12] Walker K.A., Ridley S.M., Lewis T., Harwood J.L. (1988). Fluazifop, a Grass-Selective Herbicide Which Inhibits Acetyl-CoA Carboxylase in Sensitive Plant Species. Biochem. J..

[13] Juhász C., Hadházy A., Abido W.A.E., Pál V., Zsombik L. (2024). Effect of different herbicides on development and productivity of sweet white lupine (*Lupinus albus* L.). Agronomy.

[14] International Organization for Standardization (2021). Soil, Treated Biowaste and Sludge—Determination of pH.

[15] Meier U. (2001). Growth Stages of Mono- and Dicotyledonous Plants: BBCH Monograph.

[16] World Meteorological Organization (WMO) (2018). Guide to Climatological Practices.

[17] International Seed Testing Association (ISTA) (2023). International Rules for Seed Testing.

[18] (2019). Cereals—Determination of Bulk Density, Called Mass per Hectolitre—Part 1: Reference Method.

[19] (2009). Food and Feed Products—General Guidelines for the Determination of Nitrogen by the Kjeldahl Method.

[20] AOAC International (2019). Official Methods of Analysis of AOAC International.

[21] Shapiro S.S., Wilk M.B. (1965). An analysis of variance test for normality (complete samples). Biometrika.

[22] Brown M.B., Forsythe A.B. (1974). Robust tests for the equality of variances. J. Am. Stat. Assoc..

[23] Piepho H.P., Edmondson R.N. (2018). A tutorial on the statistical analysis of factorial experiments with qualitative and quantitative treatment factor levels. J. Agron. Crop Sci..

[24] Tukey J.W. (1949). Comparing individual means in the analysis of variance. Biometrics.

[25] Jolliffe I.T., Cadima J. (2016). Principal component analysis: A review and recent developments. Philos. Trans. R. Soc. A.

[26] Benesty J., Chen J., Huang Y., Cohen I. (2009). Pearson correlation coefficient. Noise Reduction in Speech Processing.

