# Supplementary material for: Interannual Climatic Variability Modulates Biostimulant and Herbicide Effects on Yield and Seed Quality of White Lupin Under Rainfed Conditions
Source: Plants (Basel). 2026 Feb 27;15(5):726. doi: 10.3390/plants15050726 (PMC12986658; doi:10.3390/plants15050726)
Supplement: Supplementary file 1 [file plants-15-00726-s001.zip › plants-4137600-supplementary.pdf]

Table S1. Monthly precipitation totals (mm) and mean air temperatures (°C) recorded during the growing seasons (March–October) of 2024 and 2025 at the Ezăreni experimental station, compared with the 1991–2020 long-term averages.

Month	Long-term mean precipitation (1991–2020) (mm)	Precipitation 2024 (mm)	Precipitation 2025 (mm)	Long-term mean air temperature (1991–2020) (°C)	Mean air temperature 2024 (°C)	Mean air temperature 2025 (°C)
**March**	29.7	58.6	48.6	3.1	7.0	8.46
**April**	29.7	35.6	23.2	10.1	13.9	11.07
**May**	26.9	60.2	149.6	16.0	16.0	13.4
**June**	28.4	58.0	21.8	19.3	22.6	20.83
**July**	43.9	36.6	71.6	21.1	24.8	23.14
**August**	55.9	36.4	19.4	19.7	24.2	21.2
**September**	82.6	154.4	35.8	14.9	18.3	18.93
**October**	69.3	43.6	114.6	10.1	10.4	20.25

Table S2**.** Two-way ANOVA results for grain yield, thousand-seed weight (TSW), and hectoliter mass (HM) based on plot-level observations (n = 24).

Trait	Source	df	F	p-value
**Yield_kg_ha**	Year	1	55.61	<0.001
	Treatment	3	7.90	0.0019
	Year × Treatment	3	8.06	0.0017
	Error	16	–	–
**TSW_g**	Year	1	434.87	<0.001
	Treatment	3	1.52	0.2380
	Year × Treatment	3	8.86	0.0011
	Error	16	–	–
**HM_kg_hL**	Year	1	528.66	<0.001
	Treatment	3	1.59	0.2130
	Year × Treatment	3	3.83	0.0300
	Error	16	–	–

Table S3. Daily mean and maximum air temperature during the 14-day period following treatment application in 2024 and 2025.

Day after treatment	Mean Temp 2024 (°C)	Max Temp 2024 (°C)	Mean Temp 2025 (°C)	Max Temp 2025 (°C)
Day 1	23.17	31.55	14.37	24.21
Day 2	20.85	30.16	8.88	15.19
Day 3	17.09	23.62	8.94	13.28
Day 4	17.31	22.87	11.84	16.56
Day 5	18.11	23.71	12.46	18.56
Day 6	20.80	29.16	13.14	19.48
Day 7	22.15	30.76	15.48	21.54
Day 8	23.96	32.55	18.11	24.96
Day 9	25.76	32.65	18.04	25.12
Day 10	23.97	31.75	15.32	22.65
Day 11	20.79	29.66	11.50	17.96
Day 12	24.18	32.56	11.56	17.92
Day 13	24.65	32.16	12.82	18.99
Day 14	21.65	30.81	13.76	20.84
Mean ± SD	21.9 ± 2.6	30.6 ± 3.2	13.8 ± 2.9	19.9 ± 3.4

Day 1 corresponds to the date of treatment application (11 June 2024 and 15 May 2025 for the respective experimental years). Values represent daily mean and maximum air temperature recorded during the subsequent 14-day period. Meteorological data were obtained from the automated weather station located at the experimental site.
